# Longitudinal FeNO monitoring in pediatric asthma: association of FeNO and its trajectory patterns with asthma control status

**DOI:** 10.1186/s13223-026-01044-y

**Published:** 2026-06-12

**Authors:** Xiaolan Ying, Yiting Chen, Lu Liu, Yufen Wu, Jiajun Yuan, Li Hong, Yong Yin

**Affiliations:** 1 Department of Respiratory Medicine, Shanghai Children’s Medical Center, School of Medicine, Shanghai Jiao Tong University, Shanghai, China; 2 School of Public Health and Medical Technology, Xiamen Medical College, Xiamen, China; 3 Department of Pediatric Pulmonology and Immunology, West China Second University Hospital, Sichuan University, Chengdu, China; 4 Shanghai Engineering Research Center of Intelligence Pediatrics (SERCIP), Shanghai Children’s Medical Center, School of Medicine, Shanghai Jiao Tong University, Shanghai, China; 5 Research Division for Health Administration and Smart Healthcare, Child Health Advocacy Institute, China Hospital Development Institute, Shanghai Jiao Tong University, Shanghai, China; 6 Department of Clinical Nutrition, Shanghai Children’s Medical Center, School of Medicine, Shanghai Jiao Tong University, Shanghai, China

**Keywords:** Fractional exhaled nitric oxide (FeNO), Asthma, Trajectory, Children

## Abstract

**Background:**

Fractional exhaled nitric oxide (FeNO) is a non-invasive biomarker of type 2 inflammation with potential for personalized asthma management. However, the longitudinal patterns of FeNO and their association with childhood asthma control remain insufficiently characterized.

**Methods:**

This cohort study enrolled children with newly diagnosed asthma from Shanghai Children’s Medical Center. Participants underwent baseline assessments and serial FeNO measurements at diagnosis and at 3-month intervals over 12 months. Asthma control was assessed at 12 months using Global Initiative for Asthma (GINA) criteria. Longitudinal trajectories of FeNO were identified using group-based trajectory modeling (GBTM). Associations of time-specific FeNO levels and FeNO trajectory group with uncontrolled asthma at 12 months were analyzed using logistic regression.

**Results:**

Among 142 children (mean age 6.92 ± 2.08 years; 71.1% male), 125 (88.0%) achieved controlled asthma and 17 (12.0%) had uncontrolled asthma at 12 months. Elevated FeNO at 3 and 12 months was significantly associated with increased odds of uncontrolled asthma after adjusting for covariates. Longitudinal FeNO levels showed two distinct trajectories: low-stable (80.3% of participants) and high-rising (19.7%). Compared with the low-stable group, the high-rising group had significantly older age, later wheezing onset, higher serum total immunoglobulin E (IgE) and eosinophil percentages (all *p* < 0.05). Children in the high-rising trajectory group had higher odds of uncontrolled asthma (OR = 5.86; 95% CI: 1.28–29.57; *p* = 0.025).

**Conclusions:**

Elevated FeNO levels during follow-up and a high-rising FeNO trajectory are associated with uncontrolled asthma at 12 months in children with newly diagnosed asthma. Longitudinal FeNO monitoring may help identify children at increased risk of poor asthma control and support more individualized asthma management.

**Supplementary Information:**

The online version contains supplementary material available at https://doi.org/10.1186/s13223-026-01044-y.

## Introduction

Asthma is a common chronic respiratory disease that imposes a substantial health burden on children [1]. It is characterized by airway inflammation, airway hyperresponsiveness, and variable airflow obstruction [2]. Effective asthma management requires not only symptom assessment and lung function testing but also monitoring of airway inflammation to inform personalized treatment strategies [2, 3]. Fractional exhaled nitric oxide (FeNO), a non-invasive biomarker of type 2 immune activation, has gained increasing attention for its potential to assess inflammatory activity and support treatment monitoring in pediatric asthma [4, 5].

However, most studies investigating FeNO dynamics following treatment initiation are short-term, typically spanning 6 months or less. In corticosteroid-naive children with atopic asthma, baseline FeNO levels were significantly higher than those in healthy controls (30.8 ± 3.0 ppb vs. 4.0 ± 0.5 ppb; *p* < 0.01), declining rapidly within 10 days of inhaled corticosteroid (ICS) therapy, and stabilizing around 40 days [6]. Other studies have also reported FeNO reductions after 6 months of ICS therapy [7, 8]. Thus, whether FeNO follows uniform or divergent trajectories during asthma treatment remains inconclusive.

Findings regarding the relationship between FeNO and asthma outcomes have been mixed. Some studies have linked elevated FeNO levels to subsequent exacerbations or poorer asthma outcomes [9–13], whereas others have reported no significant association between FeNO and asthma control status [14]. Most prior studies have evaluated FeNO at a single time point or focused on short-term changes, rather than examining heterogeneous FeNO trajectories over extended follow-up. Limited longitudinal evidence suggested that increasing FeNO was associated with subsequent hospitalization [15]. An individual patient data meta-analysis incorporating 7 randomized controlled trials further showed that a 50% FeNO increase at 3 months was associated with an 11% increase in the odds of poor asthma control at 6 months [16]. However, another individual patient data analysis demonstrated substantial within-person variability in FeNO over 3-month intervals, suggesting that changes in FeNO may not consistently reflect clinically meaningful changes in asthma status [17]. Overall, the predictive value of both baseline and dynamic FeNO measurements for asthma outcomes remains insufficiently understood, partly due to variations in study design and insufficient follow-up duration.

This study aims to identify distinct FeNO trajectories during the first 12 months of therapy after asthma diagnosis and to evaluate their association with asthma control at 12 months. Our findings may enhance the understanding of FeNO dynamics and contribute to improved asthma management strategies.

## Materials and methods

### Subjects

This cohort study enrolled children with newly diagnosed asthma at the Respiratory Department of Shanghai Children’s Medical Center between January 1, 2019, and December 31, 2020. This was an observational cohort study. FeNO measurements and clinical assessments were performed at approximately 3-month intervals as part of routine asthma follow-up care. Asthma diagnosis adhered strictly to the Global Initiative for Asthma (GINA) criteria [18]. Inclusion criteria were: (i) age > 4 years and capable of performing spirometry; (ii) first-time asthma diagnosis with no history of long-term ICS treatment. Exclusion criteria were: (i) loss to follow-up or treatment interruption (including self-adjustment of medication dose or discontinuation); (ii) additional therapies administered beyond standard asthma medications (e.g., specific immunotherapy, biologics); (iii) presence of thoracic/airway malformations, or underlying respiratory, or other diseases potentially affecting spirometry results. Our study followed the Strengthening the Reporting of Observational Studies in Epidemiology (STROBE) reporting guideline for cohort studies. This study, derived from an institutional pediatric asthma follow-up cohort [19], addressed a distinct research question focusing on longitudinal FeNO trajectories and their relationship with asthma control.

### Study design and data collection

#### Covariates at baseline

Baseline clinical data were collected from electronic medical records (EMRs), including demographics (birth date, sex, height, weight), date of diagnosis, history of wheezing, and atopy-related and inflammatory characteristics, including eczema, family history of allergic diseases, serum total immunoglobulin E (IgE) levels, and peripheral blood eosinophil percentage (EOS%).

Pulmonary function was assessed with the JAEGER MasterScreen Pneumo spirometer (Jaeger, Germany). Essential spirometry parameters measured included forced vital capacity percentage predicted (FVC% pred), forced expiratory volume in 1 s percentage predicted (FEV1% pred), forced expiratory volume in 1 s to forced vital capacity ratio (FEV1/FVC, %), forced expiratory flow at 50% of FVC percentage predicted (FEF50% pred), forced expiratory flow at 75% of FVC percentage predicted (FEF75% pred), and forced expiratory flow between 25% and 75% of FVC percentage predicted (FEF25–75% pred).

Based on baseline spirometry results, subjects were classified into different pulmonary function phenotypes: (1) Normal Pulmonary Function (NPF): FVC% pred ≥ 80%, FEV1% pred ≥ 80%, FEV1/FVC% ≥ 80%, and at least two of FEF50% pred, FEF75% pred, or FEF25-75% pred ≥ 65%. (2) Ventilatory Dysfunction (VD): at least one parameter among FVC% pred, FEV1% pred, or FEV1/FVC% < 80%. (3) Small Airway Dysfunction (SAD): FVC% pred ≥ 80%, FEV1% pred ≥ 80%, FEV1/FVC% ≥ 80%, but at least two of FEF50% pred, FEF75% pred, and FEF25-75% pred < 65%.

#### FeNO assessment

FeNO was measured at 3-month intervals, including baseline, 3, 6, 9, and 12 months, during the initial year post-diagnosis, as recommended by the ATS guidelines [20] by FeNO analyzer (Sunvow Medical Electronics Co., Ltd., Wuxi, China). Specifically, real-time analysis of exhaled single breath was performed. Children were instructed to avoid consuming nitrate-containing foods within two hours prior to the test and to refrain from physical activity, drinking water, or eating any food within one hour. Prior to the measurement, trained technicians demonstrated each step of the procedure to the children. During the test, children sat upright, wore a nose clip, and exhaled deeply to empty the lungs and sealed their lips tightly around the disposable mouthpiece of the FeNO analyzer. After taking a deep breath in, they exhaled slowly at a steady rate of 50 mL/s.

#### Outcome assessment

The primary outcome of interest, asthma control status at the 12-month follow-up, was assessed using GINA criteria [18]. Control levels were categorized as: “Controlled,” “Partly Controlled,” or “Uncontrolled.” Therefore, children categorized as partly controlled or uncontrolled were combined into the uncontrolled group. For the primary analysis, asthma control status was dichotomized: “Controlled” versus “Uncontrolled”.

### Statistical analysis

Continuous variables were summarized as mean ± standard deviation (SD) or medians with interquartile range [IQR], or geometric mean with 95% confidence interval (CI), as appropriate. Categorical variables were reported as frequencies and percentages. Between-group comparisons were performed using Student’s t test or analysis of variance for normally distributed continuous variables, the Mann–Whitney U test or Kruskal–Wallis rank-sum test for skewed continuous variables, and the chi-squared test or Fisher exact test for categorical variables, as appropriate. FeNO values were log-transformed for analysis. Between-group differences in FeNO at each time point were assessed using Welch t tests on log-transformed FeNO values.

Binary logistic regression analysis was performed to analyze the association between the log-transformed FeNO levels measured at 3-month intervals and asthma control status at 12 months follow-up. This model yielded odds ratios (ORs) with 95% CIs for the risk of uncontrolled asthma at 12 months per 1-unit increase in log-transformed FeNO. Three models were fitted for each time point. The crude model included log-transformed FeNO only. Model 1 adjusted for age, sex, and body mass index (BMI) group. Model 2 additionally adjusted for age at first wheezing episode, number of wheezing episodes before diagnosis, eczema, family history of allergic diseases, pulmonary function phenotype, total IgE, and eosinophil percentage.

Group-based trajectory modeling (GBTM) was employed to identify longitudinal FeNO trajectories during the initial 12-month treatment period [34]. A censored normal model was used for continuous outcomes. The optimal number of trajectory groups (ranging from 2 to 5) and shape of FeNO trajectories (linear, quadratic, or cubic) were determined according to the following criteria: (1) lower Bayesian information criterion (BIC) and Akaike information criterion (AIC); (2) no less than 5% of the participants within each trajectory group; and (3) average posterior probabilities greater than 0.70 for each trajectory group [21, 22]. Model parsimony and clinical interpretability were also considered in final model selection. Finally, two distinct FeNO trajectories were determined as the best-fitting model, as shown in Tables S1-3. Binary logistic regression was applied to examine the association between FeNO trajectory group and the risk of uncontrolled asthma. All statistical analysis were conducted using the R (version 4.1.3) statistical software packages. A two-sided *p* value less than 0.05 was considered statistically significant.

## Results

**Fig. 1:**
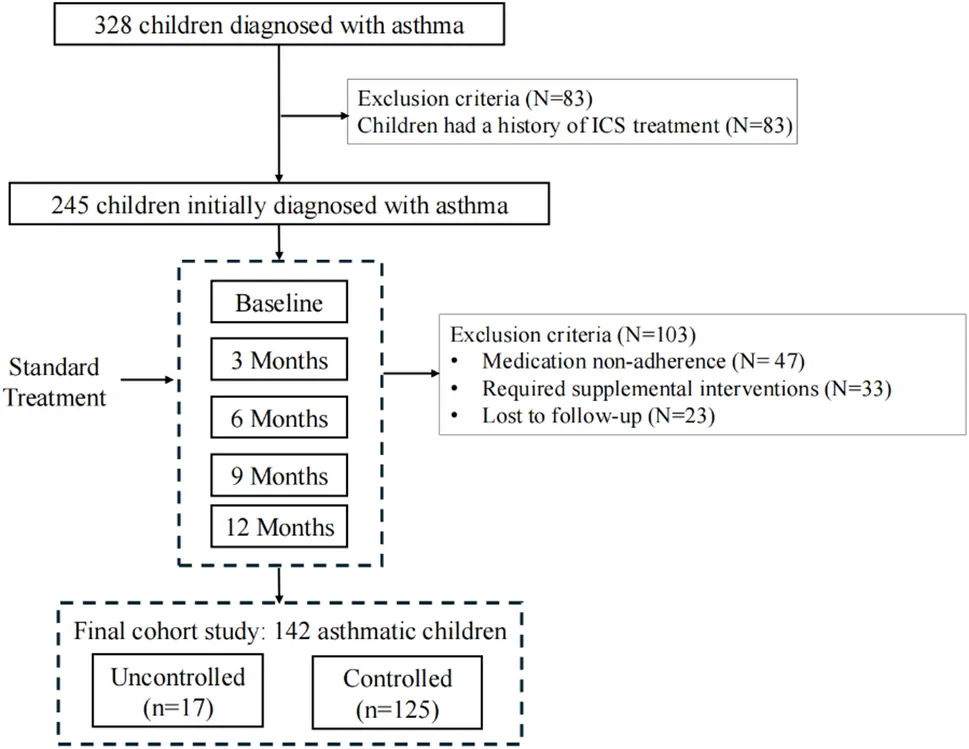
Flowchart of participants’ selection

The study initiated with 328 children diagnosed with asthma, of whom 83 with prior ICS treatment history were excluded. Of the remaining 245 children with newly diagnosed asthma, 103 were excluded during follow-up because of medication non-adherence, requirement for additional non-standard interventions, or lost to follow-up. The final analytic cohort included 142 children, of whom 125 achieved controlled asthma and 17 had uncontrolled asthma at the 12-month follow-up (Fig. 1).

The analytical cohort comprised pediatric asthma patients with a mean age of 6.92 ± 2.08 years, predominantly male (71.1%). The initial wheezing episode occurred at a mean age of 3.55 ± 2.15 years, with a median of 7 [IQR, 5.00-12.75] prior wheezing events before diagnosis. Eczema was present in 99 children (69.7%), and 119 children (83.8%) had a family history of atopy. The median serum total IgE level was 407.00 IU/mL [IQR, 181.50–655.25], and the median eosinophil percentage was 5.90% [IQR, 4.10–9.40]. Most baseline characteristics were comparable between the controlled and uncontrolled groups. The only statistically significant between-group difference was observed for FVC % pred, which was slightly higher in the uncontrolled group than in the controlled group (108.81% ± 12.51% vs. 101.06% ± 15.09%; *p* = 0.045). (Table 1).

**Table 1:** Baseline characteristics of children with asthma according to 12-month asthma control status

Variable	Overall (*n* = 142)	Controlled (*n* = 125)	Uncontrolled (*n* = 17)	*p*
Age, years	6.92 ± 2.08	6.91 ± 2.09	6.99 ± 2.11	0.884
Sex, No. (%)				0.778
Girl	41 (28.9)	37 (29.6)	4 (23.5)	
Boy	101 (71.1)	88 (70.4)	13 (76.5)	
Weight status, No. (%)				0.209
Normal	97 (68.3)	83 (66.4)	14 (82.4)	
Overweight	25 (17.6)	22 (17.6)	3 (17.6)	
Obesity	20 (14.1)	20 (16.0)	0 (0.0)	
Age at first wheezing episode, years	3.55 ± 2.15	3.58 ± 2.22	3.36 ± 1.60	0.703
Wheezing episodes before diagnosis	7.00 [5.00, 12.75]	6.00 [5.00, 12.00]	10.00 [6.00, 15.00]	0.082
Eczema, No. (%)				0.779
No	43 (30.3)	37 (29.6)	6 (35.3)	
Yes	99 (69.7)	88 (70.4)	11 (64.7)	
Family history of atopy, No. (%)				1.000
No	23 (16.2)	20 (16.0)	3 (17.6)	
Yes	119 (83.8)	105 (84.0)	14 (82.4)	
Total IgE, IU/mL	407.00 [181.50, 655.25]	409.00 [176.00, 649.00]	391.00 [219.00, 800.00]	0.528
Eosinophils, %	5.90 [4.10, 9.40]	5.90 [4.00, 9.30]	5.70 [4.70, 10.30]	0.528
Asthma phenotype, No. (%)				0.794
NPF	63 (44.4)	54 (43.2)	9 (52.9)	
SAD	51 (35.9)	46 (36.8)	5 (29.4)	
VD	28 (19.7)	25 (20.0)	3 (17.6)	
Baseline spirometric parameters				
FVC, % pred	101.98 ± 14.98	101.06 ± 15.09	108.81 ± 12.51	**0.045**
FEV1, % pred	95.96 ± 17.56	95.13 ± 17.61	102.10 ± 16.41	0.125
FEV1/FVC, %	80.01 ± 8.41	80.03 ± 8.35	79.84 ± 9.11	0.930
FEF50, % pred	68.44 ± 24.45	67.53 ± 24.01	75.06 ± 27.36	0.235
FEF75, % pred	54.52 ± 24.28	53.89 ± 24.30	59.19 ± 24.31	0.400
FEF25-75, % pred	65.50 ± 24.42	64.62 ± 24.21	71.94 ± 25.73	0.247

Bold indicates statistically significant differences between groups. FeNO, fractional exhaled nitric oxide; FVC, forced vital capacity; FEV1, forced expiratory volume in 1 s; FEF, Forced expiratory flow; IgE, immunoglobulin E; NPF, normal pulmonary function; SAD, small airway dysfunction; VD, ventilatory dysfunction

### Associations between FeNO at each time point and asthma control status at 12-month follow-up

Longitudinal FeNO levels according to asthma control status at 12 months are shown in Table 2. At 3 months, FeNO levels were significantly higher in children who subsequently had uncontrolled asthma than in those with controlled asthma (21.16 ppb [95% CI, 14.41–31.06] vs. 13.66 ppb [95% CI, 12.18–15.31]; *p* = 0.033). At 12 months, FeNO levels remained numerically higher in the uncontrolled group, although the between-group difference did not reach statistical significance (24.40 ppb [95% CI, 15.60–38.17] vs. 15.39 ppb [95% CI, 13.52–17.53]; *p* = 0.051). No significant between-group differences were observed at baseline, 6 months, or 9 months.

Table 3 showed the association between log-transformed FeNO levels at each time points and the risk of uncontrolled asthma at 12 months. At 3 months, higher FeNO levels were significantly associated with increased odds of uncontrolled asthma across all models. In the crude model, each 1-unit increase in log-transformed FeNO was associated with higher odds of uncontrolled asthma (OR, 2.66; 95% CI, 1.24–5.98; *p* = 0.014). This association remained significant after adjustment for age, sex, and BMI group (Model 1: OR, 4.17; 95% CI, 1.59–12.66; *p* = 0.006) and after further adjustment for clinical and inflammatory factors (Model 2: OR, 4.28; 95% CI, 1.48–14.81; *p* = 0.012).

At 6 months, the association was statistically significant after adjustment in Model 1 (OR, 2.70; 95% CI, 1.08–7.11; *p* = 0.037), although it did not reach statistical significance in Model 2 (OR, 2.64; 95% CI, 0.95–8.03; *p* = 0.071), suggesting a clinically relevant but attenuated trend. At 12 months, higher FeNO levels were significantly associated with increased odds of uncontrolled asthma (Model 2: OR = 2.57; 95% CI, 1.20–6.70, *p* = 0.025). Baseline and 9-month FeNO levels were not significantly associated with 12-month asthma control status (Table 3).

**Table 2:** Longitudinal FeNO levels according to 12-month asthma control status

Variable	Overall (*n* = 142)	Controlled (*n* = 125)	Uncontrolled (*n* = 17)	*p*
FeNO, geometric mean (95% CI), ppb				
Baseline	21.59 (19.33–24.13)	21.30 (19.02–23.85)	23.88 (15.24–37.41)	0.609
3 Months	14.39 (12.88–16.09)	13.66 (12.18–15.31)	21.16 (14.41–31.06)	**0.033**
6 Months	14.20 (12.81–15.73)	13.70 (12.33–15.22)	18.45 (12.58–27.05)	0.131
9 Months	16.42 (14.44–18.66)	15.93 (13.89–18.26)	20.50 (13.95–30.13)	0.208
12 Months	16.26 (14.33–18.46)	15.39 (13.52–17.53)	24.40 (15.60–38.17)	0.051

Data are presented as geometric mean FeNO levels with 95% CIs. FeNO values were log-transformed for statistical testing. Between-group differences at each time point were assessed using Welch t tests on log-transformed FeNO values. Bold indicates statistically significant differences between groups

CI, confidence interval; FeNO, fractional exhaled nitric oxide

**Table 3:** Association between FeNO and the risk of uncontrolled asthma

	Asthma uncontrolled					
	Crude model		Adjusted model 1		Adjusted model 2	
log (FeNO)	OR (95% CI)	*p*	OR (95% CI)	*p*	OR (95% CI)	*p*
Baseline	1.30 (0.61–2.85)	0.508	1.38 (0.60–3.34)	0.458	1.17 (0.47–3.04)	0.733
3 months	**2.66 (1.24–5.98)**	**0.014**	**4.17 (1.59–12.66)**	**0.006**	**4.28 (1.48–14.81)**	**0.012**
6 months	2.15 (0.95–4.97)	0.066	**2.70 (1.08–7.11)**	**0.037**	2.64 (0.95–8.03)	0.071
9 months	1.46 (0.78–2.67)	0.211	1.60 (0.79–3.20)	0.166	1.57 (0.74–3.35)	0.217
12 months	**1.98 (1.08–3.75)**	**0.027**	**2.40 (1.22–5.42)**	**0.017**	**2.57 (1.20–6.70)**	**0.025**

Odds ratios represent the odds of uncontrolled asthma at 12 months per 1-unit increase in log-transformed FeNO at each time point. Crude models included log-transformed FeNO only. Model 1 adjusted for age, sex, and BMI group. Model 2 additionally adjusted for age at first wheezing episode, number of wheezing episodes before diagnosis, eczema, family history, pulmonary function phenotype, total IgE, and eosinophil percentage. Bold indicates statistically significant differences between groups. CI, confidence interval; OR, odds ratio

**Table 4:** Comparison of Baseline Characteristics and Clinical Indicators between FeNO Trajectory Groups

	Group1: low-stable (*N* = 114)	Group2: high-rising (*N* = 28)	*p*
Age, years	6.44 ± 1.76	8.86 ± 2.20	< 0.001
Sex, No. (%)			0.650
Girl	32 (28.1)	9 (32.1)	
Boy	82 (71.9)	19 (67.9)	
Weight status, No. (%)			0.098
Normal	81 (71.1)	16 (57.1)	
Overweight	16 (14.0)	9 (32.1)	
Obesity	17 (14.9)	3 (10.7)	
Age at first wheezing episode, years	3.33 ± 1.92	4.46 ± 2.76	**0.012**
Number of wheezing episodes before diagnosis	6.00 [5.00, 12.00]	8.50 [4.75, 15.25]	0.460
Eczema, No. (%)			
No	35 (30.7)	8 (28.6)	1.000
Yes	79 (69.3)	20 (71.4)	
Family history of atopy, No. (%)			0.250
No	21 (18.4)	2 (7.1)	
Yes	93 (81.6)	26 (92.9)	
Total IgE, IU/mL	370.00 [131.25, 595.50]	560.00 [333.00, 920.25]	**0.007**
Eosinophils, %	5.60 [3.62, 9.28]	7.55 [5.77, 10.88]	**0.002**
Asthma phenotype, No. (%)			0.491
NPF	52 (45.6)	11 (39.3)	
SAD	38 (33.3)	13 (46.4)	
VD	24 (21.1)	4 (14.3)	
Baseline spirometric parameters			
FVC, % pred	101.70 ± 15.66	103.14 ± 12.03	0.651
FEV1, % pred	96.08 ± 17.82	95.46 ± 16.77	0.867
FEV1/FVC, %	80.49 ± 8.41	78.03 ± 8.24	0.165
FEF50, % pred	68.49 ± 24.34	68.19 ± 25.35	0.954
FEF75, % pred	55.34 ± 24.44	51.20 ± 23.78	0.420
FEF25-75, % pred	65.79 ± 24.19	64.28 ± 25.74	0.770
Asthma control status, No. (%)			**0.045**
Controlled	104 (91.2)	21 (75.0)	
Uncontrolled	10 (8.8)	7 (25.0)	

Bold indicates statistically significant differences between groups. BMI, body mass index; FeNO, fractional exhaled nitric oxide; FVC, forced vital capacity; FEV1, forced expiratory volume in 1 second; FEF, Forced expiratory flow; IgE, immunoglobulin E; NPF, normal pulmonary function; SAD, small airway dysfunction; VD, ventilatory dysfunction

**Table 5:** Association between FeNO trajectories and the risk of uncontrolled asthma at 12 months

FeNO Trajectories	Asthma Uncontrolled					
	Crude Model		Adjusted Model 1		Adjusted Model 2	
	OR (95% CI)	*p*	OR (95% CI)	*p*	OR (95% CI)	*p*
Group1: low-stable	ref		ref		ref	
Group2: high-rising	**3.47** **(1.15**,** 10.11)**	**0.023**	**6.55** **(1.60–29.70)**	**0.010**	**5.86** **(1.28–29.57)**	**0.025**

Crude model included FeNO trajectory group only. Model 1 adjusted for age, sex, and BMI group. Model 2 additionally adjusted for age at first wheezing episode, number of wheezing episodes before diagnosis, eczema, family history, pulmonary function phenotype, total IgE, and eosinophil percentage. The low-stable FeNO trajectory group was used as the reference group. Bold indicates statistically significant differences between groups

CI, confidence interval; OR, odds ratio

#### Associations between the trajectories of FeNO and asthma control status at 12-month follow-up

**Fig. 2:**
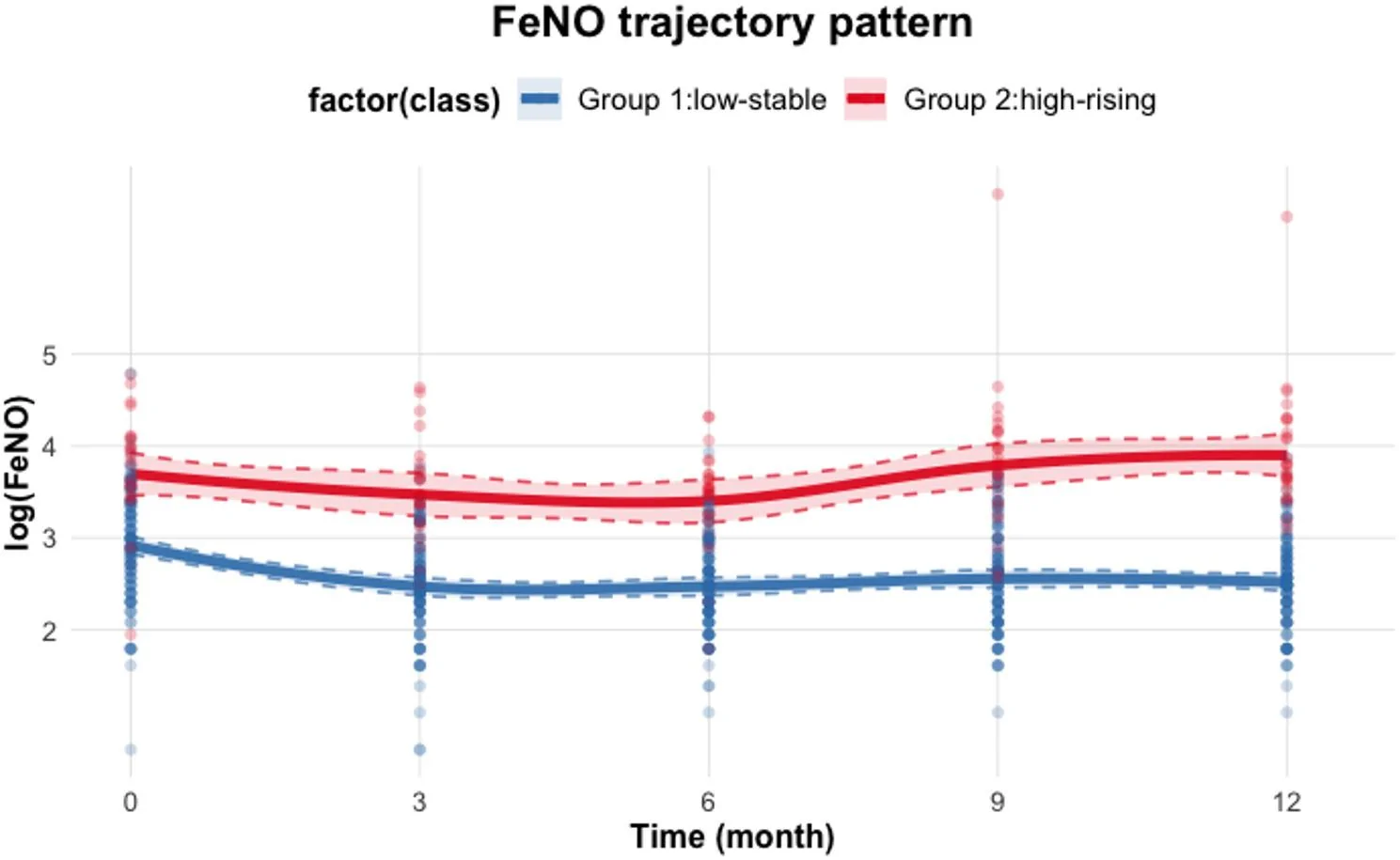
FeNO level trajectories from initial diagnosis to 12 months post-treatment

During the first year after diagnosis, two distinct trajectories of the FeNO were identified using the GBTM (Fig. 2): a low-stable pattern (114 participants, 80.3%), and a high-rising pattern (28 participants, 19.7%). As shown in Table 4, children in the high-rising group showed significantly older age than those in the low-stable group (8.86 ± 2.20 vs. 6.44 ± 1.76 years, *p* < 0.001) and had a later age at first wheezing episode (4.46 ± 2.76 vs. 3.33 ± 1.92 years, *p* = 0.012). Regarding inflammatory biomarkers, the high-rising group demonstrated significantly elevated total IgE levels (560.00 [IQR, 333.00–920.25] vs. 370.00 [IQR, 131.25–595.50] IU/mL; *p* = 0.007) and higher eosinophil percentages (7.55% [IQR, 5.77–10.88] vs. 5.60% [IQR, 3.62–9.28]; *p* = 0.002). Pulmonary function parameters and pulmonary function phenotypes were generally comparable between the two trajectory groups. Notably, the proportion of uncontrolled asthma was higher in the high-rising group than in the low-stable group (25.0% vs. 8.8%; *p* = 0.045).

The results in Table 5 showed the association between FeNO trajectory groups and the risk of uncontrolled asthma at 12 months. Compared with children in the low-stable FeNO group, those in the high-rising FeNO group had significantly higher odds of uncontrolled asthma in the crude model (OR, 3.47; 95% CI, 1.15–10.11; *p* = 0.023). After adjustment for age, sex, and BMI group in Model 1, the association remained significant (OR, 6.55; 95% CI, 1.60–29.70; *p* = 0.010). In the fully adjusted Model 2, the association remained significant (OR, 5.86; 95% CI, 1.28–29.57; *p* = 0.025) .

## Discussion

### Identification of distinct FeNO trajectories and their clinical implications

Our study identified heterogeneous longitudinal FeNO trajectories among children with newly diagnosed asthma. In particular, a high-rising FeNO trajectory was significantly associated with poorer asthma control at the 12-month follow-up. Children in this trajectory group were older and had higher eosinophils percentages and total IgE levels, consistent with established associations between FeNO and type 2 inflammation [13, 23, 24]. Age-related increases in FeNO are well documented [25], and established algorithms such as Ln(FeNO) = 1.993 + age × 0.046 further support age as an important determinant of FeNO levels [26]. This association may reflect airway growth, maturation, and age-related differences in inflammatory activity. Notably, children in high-rising group also had a later age at first wheezing episode, suggesting that delayed symptom onset may be linked to more persistent eosinophilic inflammation, possibly indicating a distinct asthma endotype.

Children with a high-rising FeNO trajectory had a higher proportion and higher odds of uncontrolled asthma at 12 months, supporting the clinical relevance of longitudinal FeNO monitoring. This finding is consistent with prior evidence linking elevated or increasing FeNO levels with worse asthma outcomes. A case-control study of 79 preschoolers with asthma and 25 healthy controls reported higher FeNO levels in those with uncontrolled asthma compared with those with controlled disease [12]. Similarly, a meta-analysis found that a 50% increase in FeNO was associated with increased odds of poor asthma control at 6 months [16]. Furthermore, children with low baseline FeNO (< 50 ppb) and minimal change over 3 months showed a lower exacerbation risk, whereas those with high baseline FeNO (≥ 50 ppb) or substantial increases experienced less stable disease [17]. Taken together, these findings support the concept that single FeNO measurements may be insufficient for risk stratification, whereas trajectory-based monitoring may provide additional information for identifying children at risk of poor control. Thus, the contribution of this study is not simply to confirm the known role of FeNO as a type 2 inflammatory biomarker, but to show that longitudinal FeNO trajectory patterns may help distinguish children with persistently or progressively elevated inflammatory activity during routine follow-up.

### FeNO as an independent predictor of asthma control

Our data align with previous research demonstrating that elevated FeNO at 3 and 12 months is associated with poorer asthma control, consistent with studies linking high FeNO to an increased risk of exacerbations and hospitalizations [9, 13]. The association at 3 months is clinically relevant because it suggests that early follow-up FeNO may help identify children whose asthma is less likely to be controlled at 12 months. The association at 12 months may reflect concurrent airway inflammation among children who remained uncontrolled. However, not all studies have observed a consistent association between FeNO and asthma control [14, 27]. For example, a prospective study of preschool children aged 3–6 years on asthma therapy indicated that, whereas baseline FeNO showed no significant difference between children who later achieved asthma control and those who did not, impulse oscillometry indices of small airway function exhibited stronger predictive performance [14]. The discrepancies between these studies and our findings are likely due to differences in study design, age range, asthma phenotype, treatment status, adherence, outcome definitions, timing of FeNO measurement, and follow-up duration. These differences suggest that FeNO should be interpreted as part of a broader clinical assessment rather than as a stand-alone marker.

### Heterogeneity of FeNO responses and potential endotypes

Persistently high FeNO levels in some children despite treatment underscores the heterogeneity of inflammatory endotypes in pediatric asthma. Previous studies have shown that FeNO levels often decrease after inhaled corticosteroid therapy [7, 8]. For example, a study involving 133 children with bronchial asthma showed that after standardized ICS treatment for 6 months, FeNO levels significantly decreased in the glucocorticoid group [7]. Another study with 247 children found that FeNO levels decreased after treatment across different medication groups, including salmeterol/fluticasone propionate, budesonide, and salbutamol [8]. Nevertheless, in a 95-patient cohort, approximately 19% maintained elevated or even increasing FeNO levels over time [15]. This pattern may indicate ongoing type 2 airway inflammation, variable treatment response, poor adherence, environmental exposures, or a phenotype requiring closer monitoring. Our findings, which also identify heterogeneous FeNO trajectories among children undergoing treatment, support the need for phenotype-informed asthma management and suggest that repeated FeNO monitoring may help identify children who require closer follow-up or treatment reassessment [28]. Several mechanisms may explain a high-rising FeNO pattern, including persistent type 2 airway inflammation, variable responsiveness to inhaled corticosteroids, suboptimal adherence, incorrect inhaler technique, ongoing allergen or environmental exposures, or comorbid allergic disease. Although our study could not directly distinguish among these mechanisms, the higher total IgE and eosinophil levels in the high-rising group support a stronger type 2 inflammatory profile.

## Strengths and limitations

This study has several strengths. First, it focused on children with newly diagnosed asthma and included repeated FeNO measurements over a 12-month follow-up period. Second, we applied group-based trajectory modeling to characterize longitudinal FeNO patterns, rather than relying only on baseline or single-time-point FeNO measurements. Third, we evaluated the association between FeNO trajectories and asthma control while considering relevant clinical, inflammatory, and pulmonary function characteristics.

Certain limitations should also be acknowledged. First, this was a single-center observational cohort study, which may limit the generalizability of the findings. However, the cohort was derived from a specialized pediatric asthma center with relatively standardized diagnosis, treatment, and follow-up assessment; future multicenter studies are needed for external validation. Second, although FeNO measurements were obtained during routine follow-up, unmeasured factors such as environmental exposures, inhaler technique, medication adherence, and day-to-day variation in FeNO may have influenced FeNO levels. Nevertheless, repeated measurements at 3-month intervals allowed us to characterize longitudinal FeNO patterns and partially reduce reliance on a single potentially variable measurement. Third, the sample size of the uncontrolled group was relatively small, which may have reduced statistical power and contributed to wide confidence intervals in regression analyses. Therefore, the magnitude of the observed associations should be interpreted cautiously. Fourth, the 12-month follow-up period provides useful medium-term evidence but does not capture longer-term FeNO dynamics, including changes after treatment step-down or discontinuation. Nonetheless, the first year after diagnosis is clinically important for treatment adjustment and early risk stratification. Finally, additional biomarkers such as sputum eosinophils, periostin, or cytokine profiles were not available, limiting the ability to further characterize underlying inflammatory endotypes. However, routinely available type 2 inflammatory indicators, including total IgE and peripheral blood eosinophil percentage, were included. Future studies with larger multicenter cohorts, longer follow-up, detailed adherence and exposure assessment, and more comprehensive biomarker profiling are needed to validate these findings and determine whether interventions guided by FeNO trajectories can improve asthma outcomes.

## Conclusions

In children with newly diagnosed asthma, elevated FeNO levels during follow-up and a high-rising FeNO trajectory were associated with uncontrolled asthma at 12 months. Routine longitudinal FeNO monitoring may help identify children at increased risk of poor asthma control. Incorporating FeNO trajectories into pediatric asthma assessment may support earlier risk stratification, phenotype-informed treatment reassessment, and more individualized asthma management.

## Supplementary Information

Below is the link to the electronic supplementary material.

Supplementary Material 1

## Data Availability

The datasets analyzed during this present study are available from the corresponding author on reasonable request.

## Abbreviations

FeNO: Fractional exhaled nitric oxide
GINA: Global Initiative for Asthma
GBTM: Group-based trajectory modeling
ICS: Inhaled corticosteroid
STROBE: Strengthening the Reporting of Observational Studies in Epidemiology
EMRs: Electronic medical records
IgE: Immunoglobulin E
EOS%: Eosinophil percentage
NPF: Normal Pulmonary Function
VD: Ventilatory Dysfunction
SAD: Small Airway Dysfunction
SD: Standard deviation
BIC: Bayesian information criterion
AIC: Akaike information criterion
IQR: Interquartile range
CI: Confidence interval
ORs: Odds ratios
BMI: Body mass index
FVC: Forced vital capacity
FEV1: Forced expiratory volume in 1 s
FEF: Forced expiratory flow
