# Supplementary material for: Longitudinal FeNO monitoring in pediatric asthma: association of FeNO and its trajectory patterns with asthma control status
Source: Allergy Asthma Clin Immunol. 2026 Jun 12;22:49. doi: 10.1186/s13223-026-01044-y (PMC13560344; doi:10.1186/s13223-026-01044-y)
Supplement: Supplementary file 1 [file 13223_2026_1044_MOESM1_ESM.docx]

**Table S1.** Model Comparison for Group-Based Trajectory Models

| Number of Classes | BIC | AIC | Classification Distribution (%) | | | | |
| --- | --- | --- | --- | --- | --- | --- | --- |
|  |  |  | 1 | 2 | 3 | 4 | 5 |
| 2 | 1309.10 | 1267.72 | 80.28 | 19.72 | NA | NA | NA |
| 3 | 1284.92 | 1225.8 | 19.72 | 0.7 | 79.58 | NA | NA |
| 4 | 1302.16 | 1225.31 | 0.7 | 15.49 | 76.06 | 7.75 | NA |
| 5 | 1329.70 | 1235.11 | 17.61 | 31.69 | 40.14 | 1.41 | 9.15 |

Models were fitted using log-transformed FeNO values measured at baseline, 3 months, 6 months, 9 months, and 12 months. Lower AIC and BIC values indicate better model fit. Classification distribution indicates the percentage of participants assigned to each trajectory group based on maximum posterior probability. The final trajectory model was selected by considering model fit, parsimony, posterior classification performance, minimum group size, and clinical interpretability. Abbreviations: AIC, Akaike information criterion; BIC, Bayesian information criterion.

**Table S2.** Parameter Estimates From the 2-Class Cubic FeNO Trajectory Model

| Group | Parameter | Estimate | Standard error | *p* |
| --- | --- | --- | --- | --- |
| Low | Intercept | 2.93 | 0.06 | <0.001 |
|  | Linear | -0.24 | 0.04 | <0.001 |
|  | Quadratic | 0.04 | 0.01 | <0.001 |
|  | Cubic | -0.002 | 0.001 | <0.001 |
| High | Intercept | 3.65 | 0.17 | <0.001 |
|  | Linear | -0.21 | 0.09 | 0.011 |
|  | Quadratic | 0.04 | 0.02 | 0.030 |
|  | Cubic | -0.002 | 0.001 | 0.093 |

Parameter estimates were obtained from the 2-class cubic group-based trajectory model using log-transformed FeNO as the outcome. The model included intercept, linear, quadratic, and cubic terms for time. P values were calculated from Wald tests. Abbreviations: FeNO, fractional exhaled nitric oxide.

**Table S3.** Parameter Estimates From the 3-Class Cubic FeNO Trajectory Model

| Group | Parameter | Estimate | Standard error | *p* |
| --- | --- | --- | --- | --- |
| 1 | Intercept | 3.65 | 0.14 | <0.001 |
|  | Linear | -0.14 | 0.08 | 0.085 |
|  | Quadratic | 0.02 | 0.02 | 0.173 |
|  | Cubic | -0.001 | 0.001 | 0.330 |
| 2 | Intercept | 3.84 | 0.45 | <0.001 |
|  | Linear | -1.72 | 0.38 | <0.001 |
|  | Quadratic | 0.37 | 0.08 | <0.001 |
|  | Cubic | -0.02 | 0.004 | <0.001 |
| 3 | Intercept | 2.93 | 0.06 | <0.001 |
|  | Linear | -0.25 | 0.04 | <0.001 |
|  | Quadratic | 0.04 | 0.008 | <0.001 |
|  | Cubic | -0.002 | 0.0004 | <0.001 |

Parameter estimates were obtained from the 2-class cubic group-based trajectory model using log-transformed FeNO as the outcome. The model included intercept, linear, quadratic, and cubic terms for time. P values were calculated from Wald tests. Abbreviations: FeNO, fractional exhaled nitric oxide.
